# The prevalence and socio-behavioural and clinical covariates of oral health related quality of life in Ugandan mothers with and without HIV-1

**DOI:** 10.1186/s12955-021-01844-3

**Published:** 2021-08-23

**Authors:** Nancy Birungi, Lars Thore Fadnes, Ingunn Marie Stadskleiv Engebretsen, James Kashugyera Tumwine, Anne Nordrehaug Åstrøm, ANRS 12174 and 12341 study groups

**Affiliations:** 1 Department of Clinical Dentistry, University of Bergen, Bergen, Norway; 2 Department of Global Public Health and Primary Care, University of Bergen, Bergen, Norway; 3 Department of Addiction Medicine, Haukeland University Hospital, Bergen, Norway; 4 Department of Global Public Health and Primary Care, Centre for International Health, University of Bergen, Bergen, Norway; 5 Department of Paediatrics’ and Child Health, School of Medicine, College of Health Sciences, Makerere University, Kampala, Uganda

**Keywords:** Dental caries, Oral health, Quality of life, HIV, Oral impacts on daily performances

## Abstract

**Background:**

There is limited evidence regarding oral health related quality of life of HIV positive populations in sub-Saharan Africa. Focusing HIV positive- and HIV negative Ugandan mothers, this study assessed the influence of HIV status on oral health related quality of life in terms of oral impacts on daily performances, whilst adjusting for clinical- and socio-behavioural factors. We also examined whether any association of clinical and socio-behavioural factors with oral impacts on daily performances vary according to mothers’ HIV status.

**Methods:**

This cross-sectional study used data from a trial (n = 164) and a comparison group (n = 181). The trial comprised of mothers with HIV-1 participating in the ANRS 121741-PROMISE-PEP-trial (NCT00640263) conducted between 2009 and 2013 and from the ANRS 12341-PROMISE-PEP-M&S follow-up study conducted in 2017. The comparison group comprised of HIV negative mothers recruited in 2017. Interviews and clinical oral examinations were performed. The oral health related quality of life was assessed using the oral impacts on daily performances frequency scale. Caries experience and gingival bleeding were assessed using the World Health Organization’s Decayed, Missed and Filled teeth indices and community periodontal index. Logistic and negative binomial regression analyses were performed.

**Results:**

29% of HIV-1 positive and 32% among the comparison reported any oral impact on daily performance. In adjusted logistic regression analysis, HIV status was not significantly associated with oral impacts on daily performances. Mother’s self-reported oral health, caries experience, gingival bleeding and oral health related quality of life of their children were independently associated with oral impacts on daily performances. Corresponding prevalence ratios and 95% confidence intervals were: 0.3 (0.2–0.6), 1.8 (1.0–3.2), 1.1 (1.0–1.1), and 2.1 (1.1–4.3). No significant interaction between HIV status and covariates were observed.

**Conclusions:**

Oral health related quality of life was substantially impaired in Ugandan mothers but did not discriminate between HIV positive and negative participants. Mothers with impaired oral health related quality of life were more likely to have dental caries and children with impaired oral health related quality of life. HIV positive and negative mothers in Uganda deserve special attention regarding their oral disease and quality of life status.

**Supplementary Information:**

The online version contains supplementary material available at 10.1186/s12955-021-01844-3.

## Introduction

The global availability and scale-up of antiretroviral treatment (ART) over the last 20 years has increased the life expectancy of persons living with the human immune virus (PLHIV) [1]. Thus, focus of treatment has shifted from opportunistic infections caused by a failing immune system to managing chronic non-communicable diseases that could occur in one’s lifetime. Globally, there are 38 million PLHIV and of them 20.7 million live in eastern and southern Africa [1]. With further scale up of the access to ART the former and later figures are expected to grow.

According to the latest global burden of disease studies (GBD), dental caries and periodontitis are non-communicable diseases, recognized to be among the leading causes of disabilities in many countries [2]. Thus, about 3.5 billion people worldwide live with dental diseases whereas 35% (2.5 billion) present with untreated caries in the permanent dentition. Moreover, severe periodontitis has been reported to be the sixth -most prevalent condition affecting 10.8% (743 million) of the global population. Corresponding figure for tooth loss is 2.3% (158 million).

There has been a growing recognition of subjective health indicators, such as the health-related quality of life (HRQoL) and oral health related quality of life [3–5]. Oral health related quality of life (OHRQoL) measures or socio-dental indicators have proved to be valid instruments to assess oral health as well as the quality of care or services of populations or groups of people [4]. Unlike clinical indicators, OHRQOL measures are subjective and capture multiple dimensions and consequences of oral disease. The dimensions are not limited to the physical consequences of disease but extend to emotional, social, financial and psychological aspects as well. A frequently used socio-dental indicator is the oral health impact on daily performances (OIDP) instrument; developed to assess oral impacts that seriously affect a person's daily life activities [6]. The OIDP assesses outcomes of disability, handicap aspects and social disadvantages of oral conditions which are part of the broader World Health Organisation (WHO)’s International Classification of Impairments, Disabilities and Handicaps (ICIDH) [7]. The ICIDH enables empirical exploration of links between different dimensions of the following key concepts, impairments, functional limitations, pain and discomfort, disability, and handicap. Locker et al. [8] amended ICIDH for dentistry. The OIDP Scale consists of 8 items that cover physical, psychological, and social dimensions of daily living. The frequency OIDP scale has demonstrated acceptable psychometric properties in the context of an oral health survey among Ugandan adolescents. Thus, discriminant and construct validity were demonstrated in that the OIDP scores varied systematically in the expected direction with missing teeth and self-report indicators of oral health status, respectively [9]. This scale has also been used to assess the impact on oral health related quality of life of periodontal disease focusing pregnant women in Uganda [10].Oral health related quality of life has been assessed among people living with HIV [11]. Australian studies, conducted before and after the implementation of the mass scaleup of ART, have reported more social impacts of oral diseases in HIV positive adults compared to HIV negative peers [12, 13]. Accordingly, a US study revealed that women with HIV had on average 10% poorer OHRQoL than uninfected women in a control group when adjusting for confounding behavioural- and clinical factors [14]. In addition, a study from Portugal reported that HIV status had a negative impact on OHRQOL after adjustment of the number of decayed teeth, prosthodontic treatment needs and drug use [15]. Tomar et al. [16] identified socio-demographic covariates of OHRQoL in terms of sex, race/ethnicity, living situation (such as prison or other institutional settings) and smoking status among patients with HIV receiving primary care in South Florida. Similar findings are reported in a recent study of US women [17]. In two Brazilian and one Malaysian study, OHRQOL of PLHIV were independently associated with general HRQOL) [18, 19]. Another Brazilian study revealed that functional and psychosocial oral impacts were weakly correlated with general quality of life measures and concluded that the functional and psychosocial oral impacts and the general HRQOL scores represented different constructs [20]. It is also evident that parents self-perceived- and objective oral health is associated with that of their offspring [21–23]. However, the association of parent’s quality of life with children’s quality of life is not yet established.

Studies considering OHRQoL among adults living with HIV have most frequently been conducted in high-and middle-income countries without inclusion of a HIV negative control group [11, 13, 15, 17, 20, 24–26]. Few studies have evaluated impacts of HIV status on OHRQoL in the context of clinical and socio-behavioural factors of adult populations living in sub-Saharan African cultural contexts. Given the huge burden of HIV in Sub Saharan Africa and the increased life expectancy of PLHIV, information regarding OHRQOL may inform a holistic management of PWHIV.

Focusing HIV positive- and HIV negative Ugandan mothers, this study assessed the influence of HIV status on OHRQoL in terms of oral impacts on daily performances, OIDP, whilst adjusting for clinical- and socio-behavioural factors. We also examined whether any association of clinical and socio-behavioural factors with OIDP vary according to mothers’ HIV status.

## Methods

This cross-sectional study compares participants from a trial cohort- with a comparison group. The trial cohort included women with HIV-1 residing in the Ugandan site (Mbale, Eastern Uganda) of the multi-center efficacy trial ANRS12174 PROMISE- PEP trial (ClinicalTrials.gov, number NCT00640263). In (that) trial, HIV-1 peri-exposure prophylaxis with ritonavir boosted lopinavir- (LPV/r) or Lamivudine (3TC), respectively, were given to the infants of the mothers who were HIV-1 positive at recruitment. The mothers got standard care and no intervention themselves. All mothers with HIV were diagnosed with the subtype HIV-1. The trial conducted between 2009 and 2013, included pregnant women with HIV-1, recruited at gestational age of 28–40 weeks at antenatal clinics in four African sites. Inclusion criteria for mother was age 18 years or older, intention to continue breastfeeding, and being HIV-1 positive, and not being eligible for ART [either clinically or because CD4 (cluster of differentiation 4) count > 350 cells/μL at that time)]. All eligible mothers and infants followed the routine prevention of mother to child (PMTCT) with antepartum zidovudine (ZDV), intrapartum nevirapine (NVP), zidovudine-lamivudine (ZDV/3TC) for mothers and NVP for infants 7 days postpartum. The ANRS12174 PROMISE- PEP trial is described in detail in a previous paper [27].

In 2017, 244 out of 278 mothers with HIV-1 infection and their HIV exposed uninfected children, HEU, were eligible for re-enrolment in the follow-up study: the PROMISE-PEP Mechanism Safety study (PROMISE-PEP M&S ANRS12341). A total of 67% of the eligible cohort of HIV-1 positive mothers (164/244) and their HEU children were followed-up leaving 32% (n = 112) lost to follow-up.

For the comparison group, 199 HIV unexposed uninfected (HUU) children, matched on age and sex with 164 HEU and their HIV negative mothers were recruited into the study. They were recruited purposively from communities located in Mbale, Eastern Uganda, which was the site for the ANRS12174 PROMISE- PEP trial. Of the 199 HUU mothers- in the comparison group, 19 were excluded due to a positive HIV-1 test result, leaving 181 HIV negative mothers enrolled. Mothers in the comparison group were tested for their HIV-1 status using serial and parallel HIV rapid testing with Determine, Stat-Pak and Uni-Gold, three test algorithms as recommended by the Ugandan Ministry of Health [28].

Thus, the present study is based on information from interviews and clinical oral examinations of 164 HIV-1 positive-mothers from the trial cohort and their 181 HIV-1 negative controls recruited into the follow up study in 2017 (mothers participating in the follow-up study from the trail cohort and 2017 recruitment of the comparison group, respectively). Mothers from these two groups are hereafter described as HIV-positive and HIV-negative mothers.

### Interviews with HIV-1 positive mothers and HIV -1 negative controls

Two trained interviewers performed face-to-face interviews with mothers using semi-structured interviews in one of the local Ugandan languages, Lumasaaba. The interview was constructed in English and translated into Lumasaba for use in the field. The instruments had been reviewed previously by project staff for semantic, experiental and conceptual equivalence of the source version. Sensitivity to culture and selection of appropriate words were considered [27, 29]. Mothers responded to questions about themselves. Information was documented on case record forms (CRFs) and electronically with Capture software System (Clinsight) and Epidata program www.epidata.dk for the clinical oral examinations.

Socio-demographic characteristics were assessed in terms of level of education, and age. Level of education was categorized into ‘did not finish primary school (1), end of primary school (2) higher education’ (3). Mothers’ perceived health was assessed as very poor (1), poor (2), fair (3), good (4), very good (5). Mothers’ perceived treatment need had the following response categories (1) not at all, (2) very little (3) to some extent, (4) considerably, (5) a great deal and recategorized to not at all/very little (0), to some extent/considerably/a great deal (1). The OHRQOL of mothers’ children was assessed using the Early Childhood Oral Health Impact Scale (ECOHIS) previously tested for its psychometric properties in the context of Ugandan pre-school children [30].

The main outcome variable, oral health-related quality of life of HIV -1 positive mothers and their negative controls was assessed using the Ugandan version of the eight-item OIDP scale previously shown to have acceptable psychometric properties among pregnant women in Uganda [10]. The OIDP questions assessed whether in the previous 6 months the respondents had problems with the following items; eating, speaking, cleaning teeth, smiling, sleeping, work performance, social contact and emotional state. For ease of interpretation in this study population, a dichotomous response scale was used in terms of no (0) and yes (1). Additive count scores, OIDP extent, were created from 8 dummy (0–8) OIDP variables, indicating number of daily activities affected. Subsequently, the OIDP extent scores (0–8) were dichotomised, yielding the categories (0) no oral impact/no daily performances affected and (1) affected on at least one daily performance/ at least one oral impact [31]. The dichotomized OIDP extent score was used in the cross-tabulation and logistic regression analyses.

### Clinical oral examination

Two experienced and calibrated dental surgeons (NB and MM) performed oral assessments among the study participants and duplicated full-mouth oral clinical examination among 26 women not included in the main study for reliability measurements. Dental caries was assessed on surface and tooth level (5 surfaces per tooth) in terms of decayed (D), missing (M), and filled (F) surface/teeth (DMFS/DMFT) in accordance with the World Health Organization (WHO) guidelines for field conditions [32]. Each surface was recoded 0 for sound and 1 for caries experience and documented as decayed if it was visually cavitated with the aid of a dental mirror and periodontal probe. A surface was recorded filled when treated and a tooth was recorded missing when extracted due to caries, as confirmed by the participant. To assess gingival bleeding of the individual, the modified community periodontal index (CPI) was used [32]. Each tooth was scored according to the presence or absence of gingival bleeding, using a periodontal probe across the gingival margins of the teeth. An individual score of ‘presence of gingival bleeding’ was given if bleeding on probing was scored for at least one tooth in the mouth.

### Statistical analysis

STATA SE 16 (College Station, Texas 77845 USA) was used for data analysis. Cross-tabulation and Chi-square tests were used with categorical variables to assess the crude associations of socio-demographic- and clinical factors and OIDP items according to HIV-1 status. The OIDP scale was used as a count (OIDP range 0–8) as well as a dichotomized outcome variable (OIDP = 0, OIDP > 0). Socio-behavioural and clinical covariates statistically significantly associated both with HIV-1 status and OIDP in unadjusted analyses were included in the logistic- and negative binomial regression models as potential confounding variables. Logistic regression was used with the OIDP as a binary outcome measure reporting prevalence ratio and 95% Confidence intervals (PR, 95% CI). Negative binomial regression was applied with OIDP as a count variable because of its skewed distribution reporting incidence rate ratios and 95% confidence intervals (IRR, 95% CI). P-values less than 0.05 were considered statistically significant. The intra examiner agreement and internal consistency of the OIDP scale were assessed using Cohen’s Kappa and Cronbach’s alpha, respectively. Pairwise interactions between socio-demographic and clinical factors and HIV status upon OIDP were included if the significance criterion set at 0.05 and 0.1 was met.

## Results

A total of 345 mothers (164 HIV positive and 181 confirmed HIV negative) completed interviews and underwent a full-mouth clinical oral examination. Cronbach’s alpha for the 8 OIDP items was 0.91. (0.90 in the HIV positive and 0.92 in HIV negative). Cronbach’s alpha of the total ECOHIS scale was 0.93. The calibration exercise comparing DMFT scores within and between examiners, revealed Cohen’s Kappa values for intra- and inter-examiner reliability of median Kappa (interquartile range) 0.7 (0.5–0.9) and 0.6 (0.4–0.8), respectively.

Table 1 show that age, type of income, self-reported oral health status, presence of gingival bleeding, caries experience, oral health related information received and child/family oral impacts according to the ECOHIS scale differed statistically significantly between HIV positive and negative mothers. The median age (25–75 percentiles) in those with and without HIV was 35 (30–40) and 31 (25–38) Above half of the participating mother were 18–32 years; 42% and 38% of respectively, HIV positive and negative mothers reported higher education. Corresponding figures regarding need for dental treatment were 13% and 13%, respectively. A higher prevalence of HIV negative than HIV positive mothers reported good oral health status (67% versus 56%) and caries experience (DMFT > 0) (81% versus 71%). Prevalence of confirmed child/family impacts according to the ECOHIS scale (ECOHIS > 0) were highest among HIV negative mothers, whereas a higher prevalence of HIV positive than HIV negative mothers confirmed having received oral health related information.

**Table 1:** Mothers’ socio-demographic-and self-reported oral health characteristics by HIV status. Percentages of those being HIV negative- and positive

	HIV negative	HIV positive	Total
	% (n)	% (n)	% (n)
Median age, years (25–75 percentiles) *Age categories*	31 (25–38)	31 (30–40)	31 (28–38)
18–32 years	57.8 (104)	39.2 (58)	49.4 (162)
33 + years	42.2 (76)	60.8 (90) **	50.6 (166)
*Education*			
End of primary	61.8 (102)	58.2 (82)	60.1 (184)
Higher than primary	38.2 (63)	41.8 (59)	39.9 (122)
*Perceived oral health*			
Poor (very poor, poor, fair)	33.3 (60)	66.7 (120)	37.8 (129)
Good (Good, very good)	66.7 (120)	57.1 (92) *	67.2 (212)
*Need for dental treatment*			
No	86.7 (156)	87.2 (143)	23.8 (71)
Yes	13.3 (24)	12.8 (21)	75.6 (34)
*Presence gingival bleeding*			
No	56.4 (102)	35.4 (58)	46.4 (160)
Yes	43.6 (79)	64.6 (106) ***	53.6 (185)
*Caries experience*			
No	28.7 (52)	58.3 (95)	24.1 (83)
Yes	71.3 (129)	81.1 (133) *	75.9 (262)
ECOHIS *-total*			
No impact	78.5 (139)	87.7 (142)	82.9 (281)
At least one impact	21.5 (38)	12.3 (20) *	17.1 (58)
*Information teeth received*			
No	73.2 (131)	58.3 (95)	66.1 (226)
Yes	26.8 (48)	41.7 (68) **	33.9 (116)

*IQR* interquartile range, *p < 0.1, **p < 0.05, ***p < 0.001

As shown in Table 2, difficulty in eating and enjoying contact with people were respectively, the most and least frequently reported impacts among both HIV positive- and negative mothers. Totals of 32.2% negative and 29.2% positive mothers reported at least one oral impact on daily performance (OIDP > 0). Among HIV positive mothers, all oral impacts discriminated statistically significantly between those reporting good and poor oral health. Among HIV negative mothers, three impacts (difficult eating, difficult speaking, difficult cleaning teeth) discriminated significantly according to their self-reported oral health status.

**Table 2:** OIDP items by global measures of oral health in HIV positive and negative mothers. Percentage of mothers with impacts. Criterion validity

	HIV POSITIVE			HIV NEGATIVE			
	Poor oral health % (n)	Good oral health % (n)	Sub-Total % (n)	Poor oral health % (n)	Good oral health % (n)	Sub-total	Total % (n)
Difficult eating	46.4 (32)	14.1 (13) **	28.0 (35)	45.0 (27)	25.0 (30*)	31.7 (57)	29.6(102)
Difficult speaking	29.0 (20)	2.2 (2) **	13.7 (22)	30.0 (18)	14.2 (17) *	19.4 (35)	16.5 (57)
Cleaning teeth	14.5 (10)	4.3 (4) *	8.7 (14)	25.0 (15)	11.7 (14) *	16.1 (289)	12.5 (43)
Sleeping	36.2 (25)	6.5 (6)	19.3 (31)	25.0 (15)	14.2 (17)	17.8 (32)	18.3 (63)
Smiling	15.9 (11)	3.3 (3) *	8.7 (14)	16.7 (10)	11.7 (14)	13.3 (24)	11.0 (38)
Emotional status	21.7 (15)	1.1 (1) **	9.9 (16)	10.0 (6)	7.5 (9)	8.3 (15)	9.0 (31)
Doing work	14.5 (10)	1.1 (1) *	6.9 (11)	8.3 (5)	5.8 (7)	6.7 (12)	6.7 (23)
Enjoying contact	10.1 (7)	1.1 (1) *	5.0 (8)	8.3 (5)	5.8 (7)	6.7 (12)	5.8 (20)
OIDP > 0	49.3 (34)	14.1 (13) ***	29.2 (47)	45 (27)	25.8 (31)	32.2 (58)	30.4 (105)
Cronbach’s alpha	0.90		0.91	0.92			

^*^p < 0.10, **p < 0.05, ***p < 0.000

As shown in Table 3, unadjusted analysis of mother’s socio-behavioural- and clinical characteristics by OIDP status revealed statistically significant associations for perceived oral health, presence of gingival bleeding, mother’s caries experience and total ECOHIS scores. When covariates significantly associated with both HIV status and OIDP were included as potential confounding variables in the multiple variable analysis, adjusted logistic regression analysis revealed that mother’s perceived oral health status, caries experience, presence of gingival bleeding and total ECOHIS score associated significantly with OIDP status. The corresponding prevalence ratios (PR) and 95% confidence intervals (CIs) were PR 0.3 (0.2–0.6); PR 1.8 (1.0–3.2); PR 1.1, (1.0–1.1); PR 2.1, (1.1–4.3), respectively. HIV status was not statistically significantly associated with mother’s OIDP. Negative binomial regression revealed corresponding incidence rate ratios (IRR) and 95% CI for for perceived oral health status were IRR 0.4 (0.2–0.7), caries experience IRR 1.8 (1.0–3.2), presence of gingival bleeding IRR1.1(1.0–1.2) and the total ECOHIS score IRR 2.3 (1.2–4.9). No statistically significant interaction between HIV status and socio-demographic and clinical covariates were observed indicating invariance of the socio-behavioural and clinical distribution of OIDP according to mothers’ HIV status (Additional file 1).

**Table 3:** Mothers’ OIDP by HIV status, socio-demographic and self-reported clinical variables. Unadjusted and adjusted OLR and negative binomial regression

	OIDP > 0 % (n)	OIDP > 0 OR (95% CI)	OIDP score range (0–8) IRR (95% CI)
*HIV status*			
Non infected	32.0 (58)	1	1
Infected	28.8 (47)	0.7 (0.4 − 1.3)	0.6 (0.3 − 1.1)
*Age*			
18–32	31.5 (50)	1	1
33 +	28.9 (48)	0.8 (0.5 − 1.4)	1.0 (0.6 − 1.7)
*Education*			
End of primary	30.4 (56)		
Higher	27.9 (34)		
*Oral health*			
Poor (very poor, poor, fair)	47.3 (61)	1	1
Good (Good, very good)	20.8 (44) ***	0.3 (0.2 − 0.6)	0.4 (0.2 − 0.7)
*Need for dental treatment*			
No	23.8 (71)		
Yes	75.6 (34) ***		
*Presence gingival bleeding*			
No	25.6 (41)	1	1
Yes	34.6 (64) *	1.8 (1.0 − 3.2)	1.8 (1.0 − 3.2)
*Caries experience*			
No	10.8 (9)	1	1
Yes	36.6 (96) ***	1.1 (1.0 − 1.1)	1.1 (1.0 − 1.2)
ECOHIS *-total*			
No impact	27.8 (78)	1	1
At least one impact	41.4 (24) **	2.1 (1.1 − 4.3)	2.3 (1.2 − 4.9)
*Information teeth received*			
No	31.9 (72)		
Yes	26.7 (31)		

^*^p < 0.10, **p < 0.05, ***p < 0.000

## Discussion

To our knowledge, this is the first study to estimate the prevalence as well as social- behavioural-, and clinical covariates of oral health related quality of life in Ugandan mothers living with HIV and their negative comparisons. The findings revealed that OHRQoL was substantially impaired in Ugandan mothers but did not discriminate between HIV positive and HIV negative participants. Neither was the socio-behavioural and clinical distribution of oral health related quality of life variant across HIV positive and negative mothers. Contrary to our expectations, the prevalence of oral impacts was about four percentage points higher in HIV negative than in HIV positive mothers regarding the total OIDP score and across the single OIDP items as well. The direction of this difference remained unchanged after adjusting for social-, behavioural-, and clinical covariates of OIDP that also varied between HIV positive- and negative mothers. The absence of significant difference in OIDP between the two groups of mothers investigated was probably not a consequence of negative confounding. In this study, OIDP items discriminated significantly according to mothers’ self-reported oral health status indicating satisfactory conceptual validity across the HIV positive- and negative mothers investigated.

A strength of this study is the inclusion of a comparison group of HIV negative mothers which further strengthens the observed findings and is contrary to most previous studies lacking a separate control group. Another strength is the use of a validated OIDP scale previously reported to be appropriate in the Ugandan context [9, 33]. The main limitation of this the study may have been loss to follow-up from the original cohort of mothers living with HIV which might limit the interpretation of the findings as the HIV cohort may suffer from being underpowered. However as reported previously [34], the analysis comparing baseline characteristics of HIV positive mothers followed-up and those lost to follow-up showed similar distributions. In terms of generalisability, the mothers with HIV in this study may be different from the population of HIV positive mothers because they were recruited to participate in a trial. However, in terms of accessing the health care system, the mothers participating in this study might be representative of Uganda mothers with HIV as their contact with the health care system is similar.

Systematic reviews have found that pregnant women had poor knowledge and awareness of oral health [35, 36]. Bindu et al. [37] also found that pregnant women placed lower priority for oral and fear for dental services were reasons for delaying dental services. Also, higher parity has been associated with number teeth present, functional tooth units of natural teeth regardless of socio-demographic and behavioural variables [38, 39]. Women of lower parity in Nigeria tended to have better oral health [40]. This study did not assess factors related to pregnancy, parity, psychosocial related factors such as attitude knowledge, attitude, and behaviour thus the absence their assessment could have limited the findings. Future studies should therefore regard the mentioned factors.

Overall, the high prevalence of OIDP observed among the participants in this study, was lower than that reported earlier among Sudanese dental patients amounting to 33.3% and 18.7% among HIV positive and HIV negative participants from the general population having the same age distribution [41], but similar to the prevalence observed in the general population of pregnant women in Uganda (i.e. 25% and 30% in urban and rural areas) [10]. Other studies emanating from adult populations in industrialized and non-industrialized countries have reported prevalence rates of OIDP ranging from 18.3% to 75% in Norway and Malaysia, respectively [42–46].

As shown in Table 2, and in accordance with previous reports from sub-Saharan Africa [10, 47–49], physical aspects in terms of difficulty with eating, speaking and sleeping were the impacts most frequently reported in both groups of mothers investigated. A recent review of HIV patients showed, accordingly that the most affected aspects of oral health related quality of life were problems with eating followed by social disability in terms of difficulty doing usual job and being irritated that were less important [11]. In contrast, HIV positive people and individuals belonging to other marginalized groups such as drug abusers living in different cultural contexts have emphasized social impacts of their oral diseases as most disabling [13].

Independent of HIV status, mothers who perceived good oral health status were less likely whereas those who presented with gingival bleeding and dental caries experience were marginally more likely to report any oral impacts on daily performances (OIDP > 0). This confirms the clinical gradient in oral health related quality of life that has been observed across HIV positive and negative populations worldwide [10, 11, 14, 50, 51]. Although, as shown by previous studies [11, 14], the disease burden in terms of the prevalence of gingival bleeding and caries experience were higher in HIV positive compared to negative mothers, this gradient was not reflected in their reported impacts on daily performances. This draws attention to the conceptual difference between clinical- and subjective indicators of oral health [8]. Subjective experience of oral health is influenced by cultural and social aspects not captured by the single clinical measurements of dental caries and periodontal disease. In accordance with the present finding, an Australian study revealed that HIV positive men were disadvantaged with regard to social impacts of their oral diseases, and that clinical indicators in terms of dental caries and periodontal disease were better among the HIV positive men than in the comparison group of general patients [12]. Similarities in oral health related quality of life among the two groups of mothers investigated in this study might be attributed to HIV positive mothers being in good health, presenting with high CD4 count which indicate optimal immunity. Also, as per treatment protocols, PLHIV on ART have routine two weekly review visits with health workers and this consultation with the health system may explain their better health quality of life compared to the control mothers who usually get in contact with health workers in response to symptoms. In addition, the fact that HIV positive mothers reported to be more frequently informed about oral health than their control counterparts might indicate appropriate access to health- and oral health care services. A previous study from industrialized country have shown that HIV positive women reported more frequent dental care utilization than did their HIV negative counterparts [14].

In this study mothers’ OIDP- and the ECOHIS scores pertaining to children’s and the family’s perceived disadvantages due to child’s oral diseases, associated significantly both in crude and adjusted analyses. This association might be viewed as a consequence of common risk factors for both OIDP and ECOHIS scores. Previous studies have shown that mother’s caries experience- a risk factor for OIDP scores, associate with caries experience of their children, which in turn have negative impacts on children’s and the family’s quality of life [21]. According to Bandura’s social cognitive theory, children’s oral health outcomes are considered a modelling process whereby children imitate their parents’ behaviours and recognize them as valued role models [52].

Most oral health promotion programmes in the Ugandan society are implemented at school level while curative services are conducted at health facility level and as outreach activities. Given the high OIDP documented in this study it is necessary to strengthen oral health prevention or promotion awareness beyond the schools and health facilities such as the media.

## Conclusion

In conclusion, OHRQoL was substantially impaired in Ugandan mothers but did not discriminate between HIV positive and HIV negative participants. Mothers with impaired OHRQoL were more likely to have dental caries and children with impaired oral health related quality of life. HIV positive and negative mothers in Uganda deserve especial attention regarding their oral disease and quality of life status.

## Supplementary Information

**Additional file 1**.** Table S1**. Mothers’ OIDP by HIV status, socio-demographic and self-reported clinical variables. Unadjusted and adjusted OLR and negative binomial regression.** Table S2**. Evaluation of effects of interaction of covariates* with HIV exposure regressed with oral health impacts.

## Data Availability

The datasets used and/or analysed during the current study are available from the corresponding author on reasonable request.

## Abbreviations

ART: Antiretroviral therapy
CI: Confidence interval
CPI: Community Periodontal impact scale
ECOHIS: Early childhood oral health impact scale
DMFT: Decayed missing filled teeth surfaces
HAART: Highly active antiretroviral therapy
HEU: HIV exposed uninfected
HIV: Human deficiency virus
HRQOL: Health related quality of life
HUU: HIV unexposed uninfected
IQR: Interquartile range
IRR: Incidence rate ratio
OHRQoL: Inter class correlation
OIDP: Oral health impacts on daily performance
NVP: Nevirapine
OR: Odds ratio
PLHIV: Persons living with Human Immune Virus
PMTCT: Prevention of mother to child transmission
PROMISE-PEP: Promoting infant health and nutrition in Sub-Saharan Africa: Safety and efficacy of infant Peri-Exposure Prophylaxis
PROMISE-PEP-M&S: Promoting infant health and nutrition in Sub-Saharan Africa: Safety and efficacy of infant Peri-Exposure Prophylaxis to prevent HIV-1 transmission by breastfeeding-Mechanisms & Safety
WHO: World Health Organisation
ZVD: Zidovudine
3TC: Lamivudine
