# Supplementary material for: The prevalence and socio-behavioural and clinical covariates of oral health related quality of life in Ugandan mothers with and without HIV-1
Source: Health Qual Life Outcomes. 2021 Aug 23;19:201. doi: 10.1186/s12955-021-01844-3 (PMC8381561; doi:10.1186/s12955-021-01844-3)
Supplement: Supplementary file 1 — Additional file 1. Table S1. Mothers’ OIDP by HIV status, socio-demographic and self-reported clinical variables. Unadjusted and adjusted OLR and negative binomial regression. Table S2. Evaluation of effects of interaction of covariates* with HIV exposure regressed with oral health impacts. [file 12955_2021_1844_MOESM1_ESM.docx]

**Table 2: Evaluation of effects of interaction of covariates* with HIV exposure regressed with oral health impacts**

Parents age

| Variable | Incidence rate ratio | 95% confidence interval |
| --- | --- | --- |
| HIV exposure | 0.7 | 0.1–3.3 |
| Parents age | 0.9 | 0.5–1.7 |
| Parents age*HIV exposure | 1.1 | 0.4–3.0 |

Education level

| Variable | Incidence rate ratio | 95% confidence interval |
| --- | --- | --- |
| HIV exposure | 0.4 | 0.08–1.7 |
| Education level | 0.7 | 0.4–1.4 |
| Education level*HIV exposure | 1.6 | 0.6–4.4 |

Perceived oral status.

| Variable | Incidence rate ratio | 95% confidence interval |
| --- | --- | --- |
| HIV exposure | 1.1 | 0.5–2.2 |
| Perceived oral status | 0.4 | 0.2–0.8 |
| Perceived oral status*HIV exposure | 0.4 | 0.2–1.2 |

Mothers need of treatment

| Variable | Incidence rate ratio | 95% confidence interval |
| --- | --- | --- |
| HIV exposure | 0.7 | 0.4–1.2 |
| Mothers need of treatment | 6.8 | 2.6–17.6 |
| Mothers need of treatment *HIV exposure | 2.4 | 0.5–10.5 |

Gingivitis

| Variable | Incidence rate ratio | 95% confidence interval |
| --- | --- | --- |
| HIV exposure | 0.9 | 0.4–1.9 |
| Adult gingivitis | 1.8 | 1.0–3.4 |
| Adult gingivitis *HIV exposure | 0.8 | 0.3–2.1 |

Caries experience

| Variable | Incidence rate ratio | 95% confidence interval |
| --- | --- | --- |
| HIV exposure | 1.4 | 0.3–5.6 |
| Caries experience | 6.6 | 2.4–17.6 |
| Caries experience*HIV exposure | 0.5 | 0.1–2.2 |

Information of teeth

| Variable | Incidence rate ratio | 95% confidence interval |
| --- | --- | --- |
| HIV exposure | 1.4 | 0.3–5.6 |
| Information of teeth | 6.6 | 2.4–17.6 |
| Information of teeth*HIV exposure | 0.5 | 0.1–2.1 |

ECOHIS

| Variable | Incidence rate ratio | 95% confidence interval |
| --- | --- | --- |
| HIV exposure | 1.2 | 0.7–2.0 |
| ECOHIS | 2.9 | 1.4–5.9 |
| ECOHIS*HIV exposure | 0.3 | 0.08–1.0 |

Table S1 Mothers’ OIDP by HIV status, socio-demographic and self-reported clinical variables. Unadjusted and adjusted OLR and negative binomial regression

|  | OIDP>0  % (n) | OIDP>1  % (n) | OIDP>0  OR (95% CI) | OIDP score range (0-8)  IRR (95% CI) |
| --- | --- | --- | --- | --- |
| *HIV status* |  |  |  |  |
| Non infected | 32.0 (58) | 24.9 (45) | 1 | 1 |
| Infected | 28.8 (47) | 22.6 (37) | 0.7 (0.4−1.3) | 0.6 (0.3−1.1) |
| *Age* |  |  |  |  |
| 18-32 | 31.5 (50) | 22.2 (36) | 1 | 1 |
| 33+ | 28.9 (48) | 25.3 (42) | 0.8 (0.5−1.4) | 1.0 (0.6−1.7) |
| *Education* |  |  |  |  |
| End of primary | 30.4 (56) | 23.4 (43) |  |  |
| Higher | 27.9 (34) | 19.6 (24) |  |  |
| *Oral health* |  |  |  |  |
| Poor | 47.3 (61) | 38.8 (50) | 1 | 1 |
| Good | 20.8 (44) *** | 15.1 (32) *** | 0.3 (0.2−0.6) | 0.4 (0.2−0.7) |
| *Need for dental treatment* |  |  |  |  |
| No | 23.8 (71) | 15.1 (32) |  |  |
| Yes | 75.6 (34) *** | 18.4 (55) *** |  |  |
| *Presence gingival bleeding* |  |  |  |  |
| No | 25.6 (41) | 32 (20) | 1 | 1 |
| Yes | 34.6 (64) * | 27 (50) * | 1.8 (1.0−3.2) | 1.8 (1.0−3.2) |
| *Caries experience* |  |  |  |  |
| No | 10.8 (9) | 3.6 (3) | 1 | 1 |
| Yes | 36.6 (96) *** | 30.2 (79) *** | 1.1 (1.0−1.1) | 1.1 (1.0−1.2) |
| ECOHIS *-total* |  |  |  |  |
| No impact | 27.8 (78) | 21.4 (60) | 1 | 1 |
| At least one impact | 41.4 (24) ** | 32.8 (19) * | 2.1 (1.1−4.3) | 2.3 (1.2−4.9) |
| *Information teeth received* |  |  |  |  |
| No | 31.9 (72) | 26.1 (59) |  |  |
| Yes | 26.7 (31) | 18.1 (21) |  |  |

*P<0.10, **p<0.05, ***p<0.000
